# Aortic Root Replacement via Lower Hemisternotomy After an Esophageal Operation

**DOI:** 10.3400/avd.cr.21-00075

**Published:** 2021-12-25

**Authors:** Kazuhiko Uwabe, Noriyasu Masuda

**Affiliations:** 1 Department of Cardiovascular Surgery, Tokyo Women’s Medical University Medical Center East, Tokyo, Japan

**Keywords:** aortic root replacement, hemisternotomy, esophagectomy

## Abstract

A 68-year-old man with a history of esophageal resection and reconstruction by gastric tube in substernal fashion required aortic root replacement for annuloaortic ectasia and severe aortic regurgitation. The gastric tube attached closely at the manubrium of the sternum and around the xiphoid process, but it positioned leftward slightly at the body of the sternum. At the operation of the aortic root replacement, we decided the lower hemisternotomy approach to avoid injury of the gastric tube. The lower hemisternotomy to access the aortic root provides a useful alternative approach in some cases with substernal reconstruction after surgery of esophageal cancer.

## Introduction

A midline full sternotomy is a gold standard approach for cardiac or aortic surgery. However, it is hazardous in cases after esophageal cancer operation, especially in which the gastric tube or colon exists in the substernal route for esophageal reconstruction. When the gastric tube or colon positions closely behind the sternum, the possibility to injure it is supposed to be high. On the other hand, less invasive cardiac surgery has been developing recently. Here, we report a successful case of aortic root replacement after esophageal reconstruction via lower hemisternotomy approach.

## Case Report

**Figure 1:**
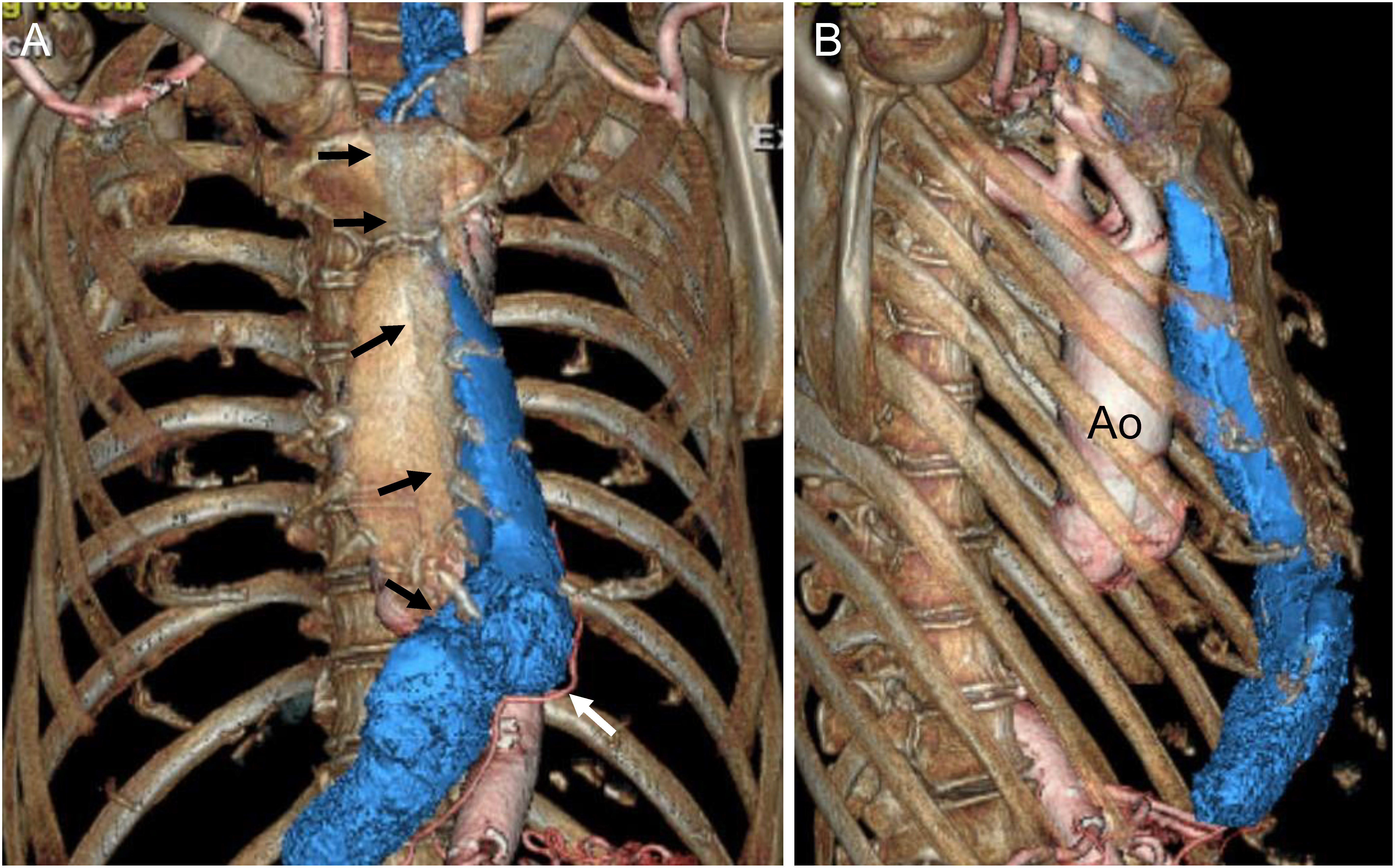
Fig. 1 Preoperative three-dimensional computed tomography. (**A**) Anterolateral view, (**B**) lateral view. The substernal gastric tube (blue) is on the midline behind the manubrium of the sternum and around the xiphoid process, but shifted to the left at the body of the sternum (black arrows). The right gastroepiploic artery located at the left side of the gastric tube (white arrow).

A 68-year-old man was referred to us for shortness of breath on exertion in 2018. He had esophageal resection due to esophageal cancer in August 2011. This operation was performed via right thoracotomy and upper midline laparotomy, and the substernal gastric tube was used for reconstruction of the esophagus. His electrocardiogram showed left ventricular hypertrophy, but the width of the cardiac shadow in chest X-ray film was within normal range. Diastolic murmur (3/VI) was heard around the fourth left sternal border. Echocardiography revealed severe aortic valve regurgitation and dilation of the sinus of Valsalva and left ventricle. Left ventricular ejection fraction was normal, and no other valvular abnormalities were noted. Aortic valve regurgitation was detected from multiple points in color Doppler echocardiogram, and these findings indicated perforations of the cusps or large fenestrations. Computed tomography demonstrated that the diameter of the sinus of Valsalva was 50 mm and the aortic valve annular diameter was 29 mm. The gastric tube for esophageal reconstruction occupied the substernal space and attached closely at the manubrium of the sternum and around the xiphoid process, but shifted leftward slightly at the body of the sternum (**Fig. 1**). It was expected that a conventional midline full sternotomy induced injury of the gastric tube. On the other hand, wide adhesion in the right thoracic cavity makes it difficult to access the aortic root via right thoracotomy. Thus, we scheduled to perform aortic root replacement via lower hemisternotomy approach.

Ao: aorta

**Figure 2:**
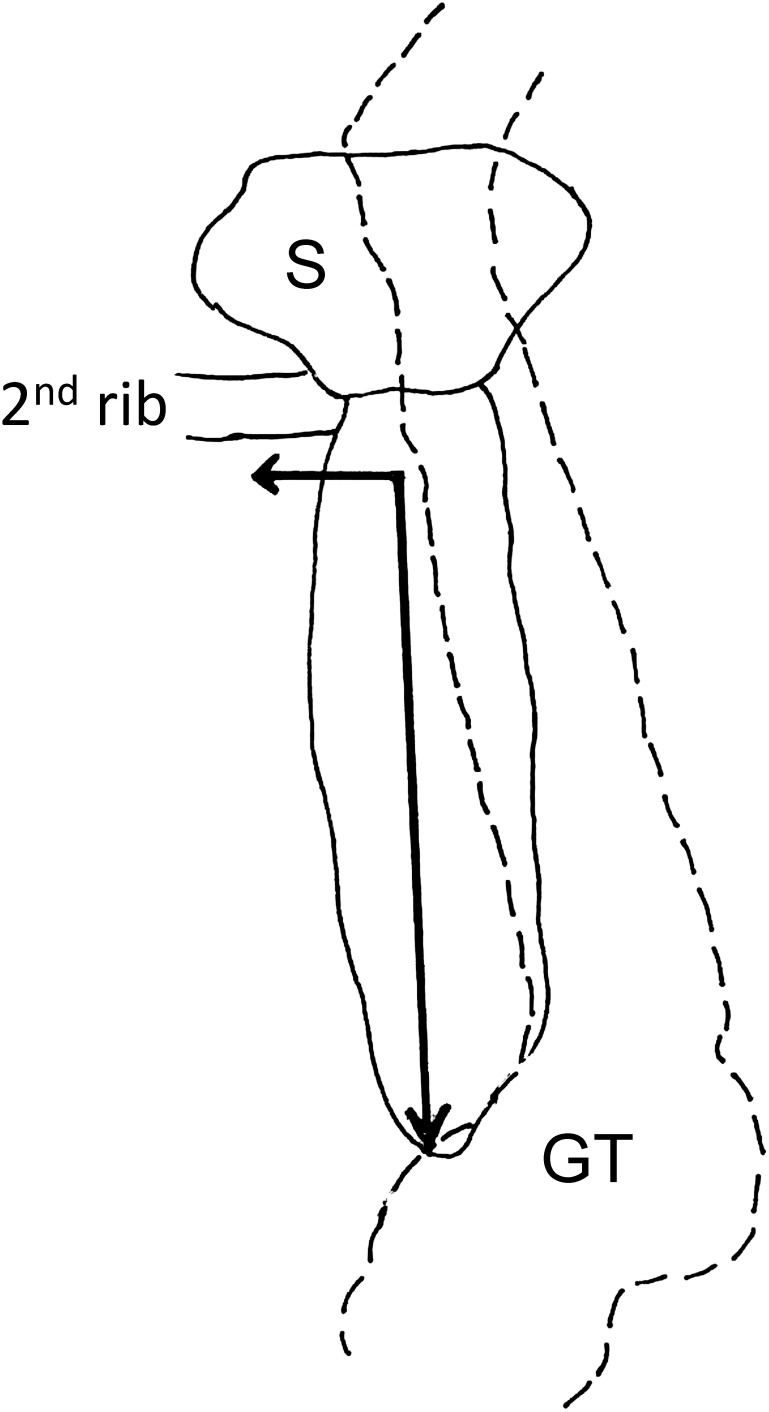
Fig. 2 Hemisternotomy. A lower hemisternotomy was made in reverse “L” fashion (arrow line segment).

**Figure 3:**
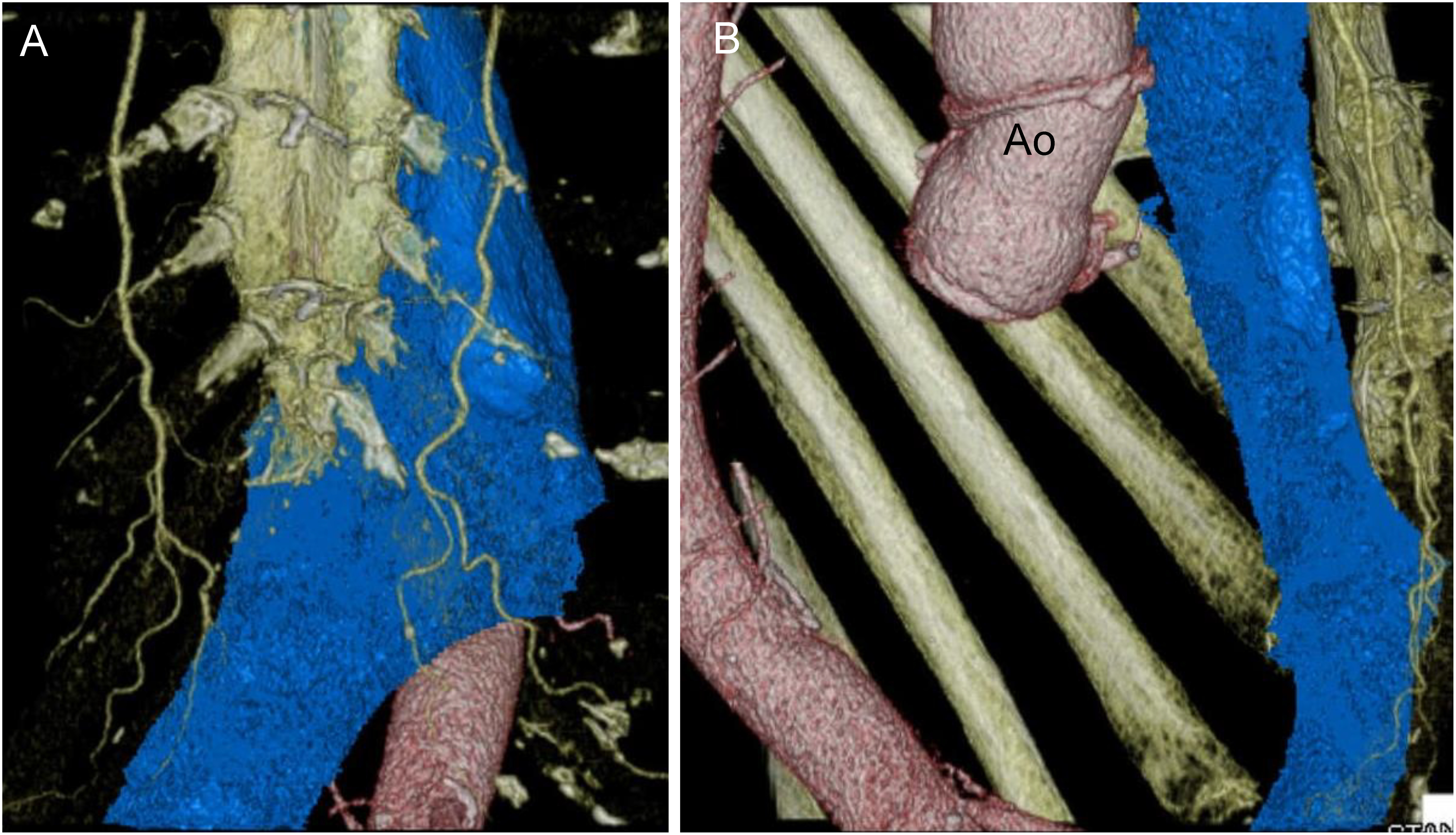
Fig. 3 Postoperative three-dimensional computed tomography. The aortic root was replaced without damage of the gastric tube.

A 9-cm midline skin incision on the lower sternum was made. The right half of the body of the sternum, only where the gastric tube shifted leftward, was divided from the second intercostal space to the sternoxiphoid junction in reverse “L” fashion (**Fig. 2**). By opening the body of the sternum, the location of the gastric tube was changed to midline. We placed the gastric tube away carefully and gently leftward and opened the pericardial sac. Several pericardial stay sutures on the left and diaphragm edges were placed to protect the gastric tube during operative maneuver. Cardiopulmonary bypass was initiated through cannulas in the right femoral artery and vein. As venous drainage was not enough, an additional venous drainage cannula was directly placed in the superior vena cava through the incision. After the ascending aortic cross-clamping, the proximal aorta was opened transversely, and cardioplegic solution was infused selectively. All the cusps had large fenestrations and appeared to be fragile. These findings of the aortic valve were equivalent to the echocardiogram, and we decided to replace the aortic valve. The aortic valve was resected, and both coronary ostia were excised from the aortic wall in button fashion. The aortic root and proximal part of the ascending aorta were replaced with a composite graft using a bioprosthesis and a woven fabric graft, and both coronary buttons were implanted to the composite graft. Weaning of cardiopulmonary bypass was smooth, and the patient had an uneventful postoperative recovery (**Fig. 3**).

S: sternum; GT: gastric tube (dashed line)

Ao: aorta

## Discussion

A midline full sternotomy is a gold standard approach for most cardiac and aortic operations. It is the same in many reoperations. However, in some cases in which the gastric tube or colon for esophageal reconstruction adheres to the sternum closely and widely after an esophageal cancer operation, an irregular approach is needed to perform cardiac and aortic operation safely.^1,2)^ Although the right thoracotomy is an alternative approach for this situation, wide adhesion in the right thoracic space is expected because of previous esophageal operation via right thoracotomy. Moreover, less invasive cardiac surgery has been developing recently.^3–6)^ Transcatheter aortic valve implantation is selected for many cases of aortic stenosis in this situation, but this maneuver is not an indication for aortic regurgitation with the dilated sinus of Valsalva. In the present case, the gastric tube attached closely at the manubrium of the sternum and at the epigastric area, but it shifted leftward slightly at the body of the sternum. It was supposed that the gastric tube would not be injured by limiting sternotomy on the body of the sternum. Therefore, we selected a lower hemisternotomy approach in reverse “L” shape for aortic root replacement to perform the operation safely.

Since blood flow of the gastric tube usually depends on the right gastroepiploic artery, surgeon must pay attention not to injure the main body of the gastric tube and adjacent left side tissue of the tube. Left side pericardial stay sutures were important to protect the gastric tube from accidental injury during operative procedure. Lower hemisternotomy approach is sometimes used in less invasive aortic valve replacement, in which the aortic valve is located more caudally than usual. This approach provided excellent exposure for aortic root and almost the whole length of the ascending aorta. We could also add a cannula into the superior vena cava through the incision to improve venous drainage, and it did not interfere with operative procedure.

In case of heavy and wide attachment of the gastric tube behind the sternum, the approach via right thoracotomy would be selected despite adhesion in the right pleural space. However, preoperative accurate measurements on the diagnostic imaging and detail estimation of other organ anatomy are able to modify the irregular operation to the near usual one. Planning of operation is especially important in atypical cases. Lower hemisternotomy in the substernal position of the gastric tube is an alternative useful approach to the aortic root.

## Conclusion

The lower hemisternotomy to access the aortic root provides a useful alternative approach in some cases with substernal reconstruction after surgery of esophageal cancer.
